# Bladder-cancer-derived exosomal circRNA_0013936 promotes suppressive immunity by up-regulating fatty acid transporter protein 2 and down-regulating receptor-interacting protein kinase 3 in PMN-MDSCs

**DOI:** 10.1186/s12943-024-01968-2

**Published:** 2024-03-09

**Authors:** Xiaojun Shi, Shiyu Pang, Jiawei Zhou, Guang Yan, Ruxi Gao, Haowei Wu, Zhou Wang, Yuqing Wei, Xinyu Liu, Wanlong Tan

**Affiliations:** 1 Department of Urology, Nanfang Hospital, Southern Medical University, Guangzhou, 510515 China; 2 Southern Medical University, Guangzhou, China

**Keywords:** PMN-MDSCs, FATP2, RIPK3, CircRNA, Bladder cancer

## Abstract

**Background:**

Polymorphonuclear myeloid-derived suppressor cells (PMN-MDSCs) is one of the causes of tumor immune tolerance and failure of cancer immunotherapy. Here, we found that bladder cancer (BCa)-derived exosomal circRNA_0013936 could enhance the immunosuppressive activity of PMN-MDSCs by regulating the expression of fatty acid transporter protein 2 (FATP2) and receptor-interacting protein kinase 3 (RIPK3). However, the underlying mechanism remains largely unknown.

**Methods:**

BCa-derived exosomes was isolated and used for a series of experiments. RNA sequencing was used to identify the differentially expressed circRNAs. Western blotting, immunohistochemistry, immunofluorescence, qRT-PCR, ELISA and Flow cytometry were performed to reveal the potential mechanism of circRNA_0013936 promoting the immunosuppressive activity of PMN-MDSC.

**Results:**

CircRNA_0013936 enriched in BCa-derived exosomes could promote the expression of FATP2 and inhibit the expression of RIPK3 in PMN-MDSCs. Mechanistically, circRNA_0013936 promoted the expression of FATP2 and inhibited the expression of RIPK3 expression via sponging miR-320a and miR-301b, which directly targeted JAK2 and CREB1 respectively. Ultimately, circRNA_0013936 significantly inhibited the functions of CD8^+^ T cells by up-regulating FATP2 through the circRNA_0013936/miR-320a/JAK2 pathway, and down-regulating RIPK3 through the circRNA_0013936/miR-301b/CREB1 pathway in PMN-MDSCs.

**Conclusions:**

BCa-derived exosomal circRNA_0013936 promotes suppressive immunity by up-regulating FATP2 through the circRNA_0013936/miR-320a/JAK2 pathway and down-regulating RIPK3 through the circRNA_0013936/miR-301b-3p/CREB1 pathway in PMN-MDSCs. These findings help to find new targets for clinical treatment of human bladder cancer.

**Supplementary Information:**

The online version contains supplementary material available at 10.1186/s12943-024-01968-2.

## Introduction

Bladder cancer is a common malignant tumor in the urinary system, and patient with invasive bladder cancer has a poor prognosis [1]. Tumor cells can evade the attack of immune killing cells through various mechanisms, which is one of the main reasons for the poor efficacy of tumor immunotherapy [2]. Polymorphonuclear bone marrow-derived suppressive cells (PMN-MDSCs) are pathological activated immune cells with strong immunosuppressive functions, which appear in many diseases [3]. PMN-MDSCs infiltrating the tumor microenvironment are one of the main culprits for tumor progression and poor immunotherapy efficacy, and their presence is significantly correlated with poor prognosis [4]. PMN-MDSCs can induce immune tolerance through overexpression of inducible nitric oxide synthase (iNOS), reactive oxygen species (ROS) and arginase 1 (Arg-1) [5, 6]. According to literature reports, the factors that induce the activation of MDSCs mainly include M-CSF, IL-6, GM-CSF, and VEGF [7]. Among various triggering factors, GM-CSF is considered a key initiating factor for the activation of PMN-MDSCs [8]. JAK protein family members and transcription activating factor (STAT) are key signaling molecules that promote the proliferation of MDSCs [9]. Although there has been some progress in the regulatory mechanism of PMN-MDSCs in recent years, the mechanism of pathological activation of PMN-MDSCs is still largely unknown, which limits the selective targeting of these cells.

Receptor interacting protein kinase 3 (RIPK3) and fatty acid transporter 2 (FATP2) are important molecules that regulate the immunosuppressive functions of MDSCs [8, 10]. FATP2 (long-chain fatty acid transporter and acetyl Coa synthase) is a key enzyme that promotes fatty acid oxidation and lipid synthesis [11]. A previous study confirmed that FATP2 enhanced the immunosuppressive function of PMN-MDSCs by promoting the synthesis of prostaglandin E2 (PGE2), and the over-expression of FATP2 in PMN-MDSCs was controlled by GM-CSF, through the activation of phospholated STAT5 transcription factor [8]. . In addition, RIPK3 is also one of the key molecules regulating tumor immunity [12, 13]. As reported by Yan et al., there is a RIPK3-PGE2 circuit in PMN-MDSCs, which can greatly promote the synthesis of PGE2 and enhance the immunosuppressive activity of PMN-MDSCs [10]. In this study, we found that there were abundant immunosuppressive PMN-MDSCs infiltrating in the tumor tissue of bladder cancer. In addition, the expression of FATP2 was up-regulated, while the expression of RIPK3 was down-regulated in bladder tumor tissue. Moreover, the infiltration of PMN-MDSCs was positively correlated with the expression of FATP2 and negatively correlated with the expression of RIPK3. However, the mechanism by which PMN-MDSCs simultaneously up-regulate FATP2 and down-regulate RIPK3 mediated suppressive immunity in the tumor microenvironment is still unclear.

Circular RNA (circRNA) is a group of endogenous non coding RNA molecules that connect from head to tail through reverse splicing to form a covalent closed loop structure [14]. More and more studies have shown that circRNA plays a crucial role in regulating tumor proliferation and metastasis in various human malignant tumors [15]. They may serve as microRNA (miRNA) sponges [16], RNA binding protein sponges and protein scaffolds [17], transcription regulators [18], or protein translation templates [19]. Due to its high stability, widespread expression, and tissue specificity, circRNA is becoming a promising cancer biomarker and therapeutic target. In our study, we found that a circRNA (hsa_circ_0013936) enriched in bladder cancer (BCa)-derived exosomes could regulate the expressions of FATP2 and RIPK3 in PMN-MDSCs. However, the specific roles and mechanism of hsa_circ_0013936 in immune regulation of bladder cancer are largely unknown. Therefore, the purpose of this study is to elucidate the potential molecular mechanism that up-regulating the expression of FATP2 and down-regulating the expression of RIPK3 in PMN-MDSCs. This study will provide new ideas for cancer immunotherapy.

## Materials and methods

### Human samples

We collected tumor tissue samples from 18 patients (12 women and 6 men) with bladder cancer admitted to South Hospital of Southern Medical University (Guangzhou, China). The patient’s age ranges from 42 to 70 years old. This study was evaluated and approved by the Ethics Committee of Southern Hospital. All patients signed informed consent forms before inclusion.

### Cell culture and transfection

Bladder cancer cell lines (T24, UMUC3, BIU-87) were purchased from the stem cell bank of the Chinese Academy of Sciences. All cell lines grew on RPMI-1640 medium containing 10% fetal bovine serum. Cells were cultured using Mycoplasma OUT to prevent Mycoplasma contamination. We identified all cell lines every six months to ensure their quality. According to the manufacturer’s instructions, cells were transfected with designated nucleotides or plasmids using Lipofectamine 3000. SiRNAs targeting circ_0013936, miR-320a, miR-301b-3p, JAK2, CREB1 and negative control (NC) siRNA were purchased from RiboBio Corporation (Guangzhou, China).

### Immunofluorescence

Tumor tissues were prepared into frozen sections and incubated with rabbit anti-mouse CD11b (eBioscience) and rat anti‐mouse LOX-1 antibodies (eBioscience) for 1 h, and then added the second antibodies (Alexa Fluid 647 labeled goat anti rabbit Abs and Alexa Fluid 488 labeled goat anti rat Abs) (eBioscience) and incubated for 30 min. After staining with Vectashield DAPI, fluorescence microscopy was used to observe CD11b^+^LOX-1^+^cells in tumor tissues.

### Immunohistochemistry

Tumor tissues were sequentially fixed, paraffin-embedded, dewaxed, rehydrated, and antigen retrieval. The samples were incubated with primary antibody (Epitomics), and then incubated with secondary biotinylated antibody. The anti-rat Ig SABC kit (spring) was used to visualize the positively expressed protein. After staining with hematoxylin, the expression of positive protein was quantified under a microscope.

### Western blot analysis

After the cells were lysed using RIPA lysis buffer, the samples were sequentially subjected to protein extraction and electrophoresis, transfer to membrane and blocking. Then the samples were incubated overnight with the primary antibody at 4 ℃, and then incubated with the second antibody coupled with horseradish peroxidase at room temperature for 60 min. After incubation with chemiluminescence, the membrane was finally visualized using a Tanon 5200 system.

### Isolation of PMN-MDSCs and CD8^+^ T-cells, and proliferation assay

PMN-MDSCs were separated by magnetic beads (BioLegend), and CD8^+^ T-cells were separated by CD8^+^ T Cell Enrichment Kit (BioLegend). Then, PMN-MDSCs and carboxyfluorescein diacetate, succinimidyl ester (CFDA-SE) labeled CD8^+^T cells were cocultured for 72 h. To count the CD8^+^CFSE^+^ and CD8^+^IFN-γ^+^ T-cell subpopulation, T-cells were first stained with FITC-labeled anti-CD8 (Biolegend), and then stained with PE-labeled anti-IFN-γ antibody (Biolegend). The CD8^+^CFSE^+^ and CD8^+^IFN-γ^+^ T-cells were detected by flow cytometry (Becton Dickinson).

### Isolation and identification of BCa-derived exosomes

Extracellular vesicles were obtained from the culture supernatant of BCa cells after four consecutive centrifugations (300×g for 5 min, 1200×g for 20 min, 10,000×g for 30 min and 110,000×g for 70 min). The concentration and size of extracellular vesicles were evaluated through nanoparticle tracking analysis (NTA). The morphology of the extracellular vesicles was observed using a transmission electron microscope (JEOL, Tokyo, Japan). The expressions of exosomal markers were detected by Western blotting. According to the manufacturer’s instructions, BCa-derived exosomes were labeled with PKH67 Green Fluorescent membrane linker dye (Sigma), and then cocultured with PMN-MDSCs. After incubation at 37 ℃ for 2 h, the cells were observed using a fluorescence microscope.

### Double luciferase reporter gene assay

We designed and obtained Olgonucleide pairs containing the required target or mutant regions from GenePharma (Shanghai, China). Double luciferase assay was performed in a 96 well plate. After 8 h of cell attachment, they were co transfected with a 50ng mimic or control. 48 h after transfection, cell lysates was harvested. According to the manufacturer’s protocol, the renilla luciferase activity was measured by a dual luciferase reporter gene assay kit.

### Quantitative real-time PCR and genomic DNA extraction

The Arcturus PicoPure RNA isolation kit (Biosciences) was used to extract total RNA. The reverse transcription system (Toyobo) was used to synthesize cDNA. SYBR Green PCR Master Mix (Applied Biosystems) was used for Real-time PCR analysis. The relative expression of target genes was calculated using the 2^−ΔΔCt^ method. Genomic DNA was extracted from cells by the Easy Pure Genomic DNA kit (Transgen Biotech).

### Nuclear‑cytoplasmic fractionation

According to the manufacturer’s instructions, the cell nucleus and cytoplasm were separated using cell nucleus and cytoplasm extraction reagents (Termo Fisher Scientific, USA). The data were calculated using 2^−ΔΔCt^ method. GAPDH was cytoplasmic control, and U6 was nuclear control.

### RNase R and actinomycin D treatment

Total RNA extracted from cells cultured and incubated with or without Ribonuclease R (Epicentre Technologies). qRT-PCR was used to detect the expression of hsa_circ_0013936 and other RNA. BCa cells were treated with actinomycin D or dimethyl sulfoxide (Sigma), and then the stability of hsa_circ_0013936 was detected by qRT-PCR.

### Fluorescence in situ hybridization (FISH)

FISH assay was performed to detect the location of hsa_circ_0013936. FISH reagent kit (RiboBio) was used to check signals according to the manufacturer’s instructions. The experimental results were visualized in the Nikon AISi laser scanning confocal microscope.

### Biotinylated RNA pull‑down assay

Biotin-labeled hsa_circ_0013936 probe synthesized by RiboBio (Guangzhou, China) was mixed with streptavidin magnetic beads (Beaver, China). Then the probes complex was incubated with the BCa cell lysate, with the oligonucleotide probe as the control. The pull-down assay was performed using PierceTM Magnetic RNA-Protein Pull-Down Kit (Termo Fisher Scientifc) according to the manufacturer’s instructions. The hsa_circ_0013936 and miRNAs were extracted by TRIzol (Invitrogen) and analyzed by RT-qPCR.

### Liquid chromatography-mass-spectrometry (LC-MS) of lipids

PMN-MDSCs were separated by magnetic beads (BioLegend). The lipid and PGE2 concentrations in the cell supernatant were detected to evaluate the lipid metabolism ability of PMN-MDSCs. The Acquity UPLC system (Waters) was used to separate extracted samples. The concentrations of Lipids and PGE2 were detected using scheduled multiple reaction monitoring (MRM). Analyst 1.6.2 software (Applied Biosystems) was used to analyzed the LC-MS data.

### ELISA

The supernatant of PMN-MDSCs was harvested and coated onto the surface of a microplate well and incubated overnight at 4 degrees Celsius to allow the molecules to adhere to the well. Sequentially undergoing blocking, sample and standard addition, primary antibody incubation, secondary antibody incubation, substrate addition, stop reaction, colorimetric detection and data analysis, the concentrations of Arg-1, IL-10 and iNOS in supernatant of PMN-MDSCs were detected using ELISA.

### Statistics

All statistical analyses were performed using SPSS version 21 (SPSS, Chicago). ANOVA and Student’s t tests were performed to analyze the differences in mean values between groups. The correlation between FATP2 and RIPK3 expression and PMN-MDSCs infiltration were analyzed using a χ2 test. *P* < 0.05 was considered statistically significant.

## Results

### Expressions of FATP2 and RIPK3 in bladder tumor tissues infiltrating PMN-MDSCs

**Fig. 1:**
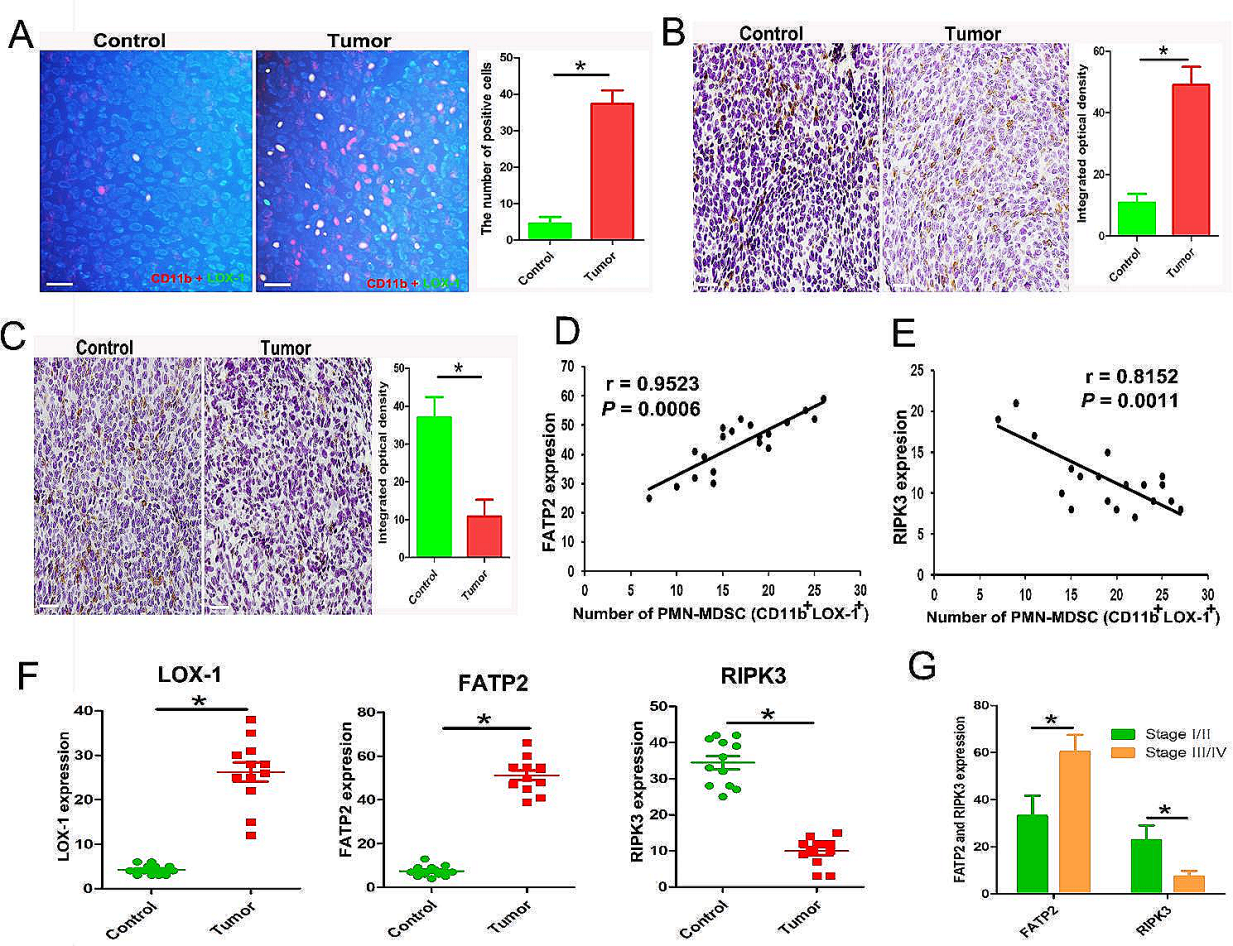
PMN-MDSCs infiltrating the tumor microenvironment with overexpression of FATP2 and low expression of RIPK3. (**A**) Detection of PMN-MDSCs (CD11b^+^LOX-1^+^) (yellow) in bladder cancer tissues and controls (adjacent tissues) by immunofluorescence (bar = 200 μm). (**B** and **C**) Immunohistochemical detection of RIPK3 and FATP2 in bladder cancer tissues and adjacent tissues (bar = 200 μm). (**D** and **E**) Correlation analysis of expression of RIPK3 or FATP2 and PMN-MDSCs infiltration in bladder tumor tissue. (**F**) Immunohistochemical detection of LOX-1, RIPK3 and FATP2 expression in human bladder cancer tissues (*n* = 18). (**G**) The expression of RIPK3 and FATP2 in bladder cancer patients with different clinical stages. (* *P* < 0.05)

Immunofluorescence was performed to detect PMN-MDSCs infiltration in tumor tissue. The double positive cells (CD11b^+^LOX-1^+^) were considered PMN-MDSCs. Immunohistochemistry was performed to display the FATP2 and RIPK3 expressions. Figure 1A shows the presence of plentiful PMN-MDSCs in tumor tissues. As shown in Fig. 1B and C, the expression of FATP2 is significantly up-regulated, and the expression of RIPK3 is significantly down-regulated in tumor tissues.The statistical analysis results indicate that the infiltration of PMN-MDSCs in bladder tumor tissue is positively correlated with the expression of FATP2 and negatively correlated with the expression of RIPK3 (Fig. 1D and E). The immunohistochemical results of human bladder cancer showed that the expressions of LOX-1 and FATP2 in human bladder tumor tissues were higher than that in paracancerous tissue, and the expression of RIPK3 in tumor tissue was lower than that in paracancerous tissue (Fig. 1F) (Supplementary Fig. 1). Additionally, compared to stage I/II patients, stage III/IV patients have high expression of FATP2 and low expression of RIPK3 in tumor tissues (Fig. 1G).

### BCa-derived exosomes up-regulated FATP2 and down-regulated RIPK3 in PMN-MDSCs

**Fig. 2:**
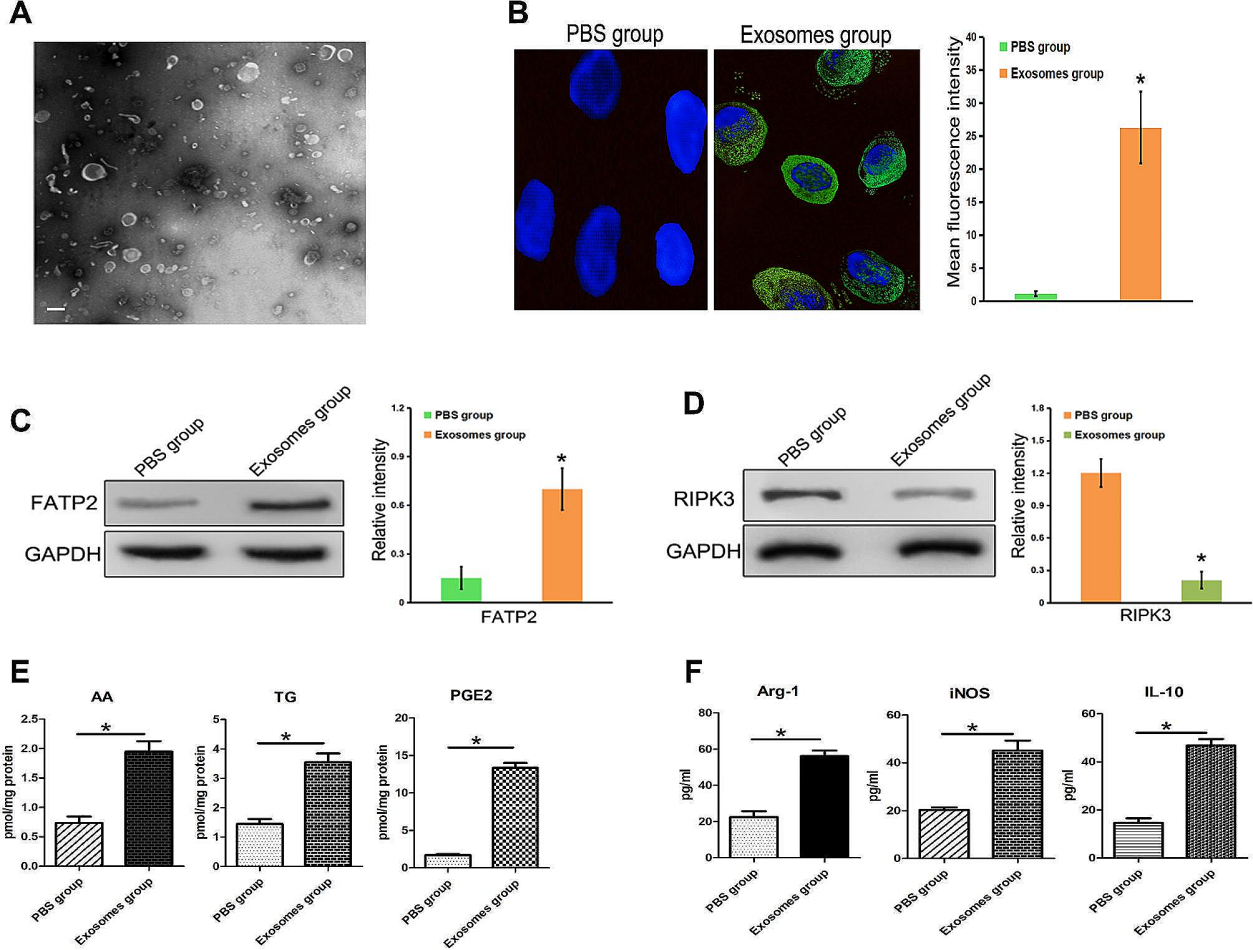
BCa-derived exosomes up-regulated FATP2 and down-regulated RIPK3 in PMN-MDSCs. (**A**) Morphological characteristics of BCa-derived exosomes revealed by transmission electron microscopy (bar = 100 nm). (**B**) Immunofluorescence showed that the BCa-derived exosomes (green) were gradually taken up by PMN-MDSCs. (**C** and **D**) Detection of the expression of RIPK3 and FATP2 in PMN-MDSCs using Western blotting. (**E**) Detection of Prostaglandin E2 (PGE2), Arachidonic Acid (AA), and Triglycerides (TG) in PMN-MDSCs by LC/MS. (**F**) ELISA detected the levels of immunosuppressive molecules (IL-10, INOS, Arg-1) in PMN-MDSCs. (*P < 0.05)

Transmission electron microscopy shows that BCa-derived exosomes have a complete and continuous bilayer membrane, which is a typical morphological feature of exosomes (Fig. 2A). After co culturing with PMN-MDSCs, BCa-derived exosomes labeled with fluorescent PKH67 were internalized by PMN-MDSCs (Fig. 2B). When PMN-MDSCs were co cultured with BCa-derived exosomes, the expression of FATP2 was significantly up-regulated and the expression of RIPK3 was significantly down-regulated in PMN-MDSCs (Fig. 2C and D). LC-MS analysis showed that BCa-derived exosomes promoted the synthesis of lipid metabolic molecules (PGE2, TG, AA) in PMN-MDSCs (Fig. 2E). In addition, ELISA analysis showed that BCa-derived exosomes promoted the synthesis of immunosuppressive molecules (IL-10, iNOS, Arg-1) in PMN-MDSCs (Fig. 2F).

### Identifcation and characterization of circRNA_0013936 in BCa-derived exosomes

**Fig. 3:**
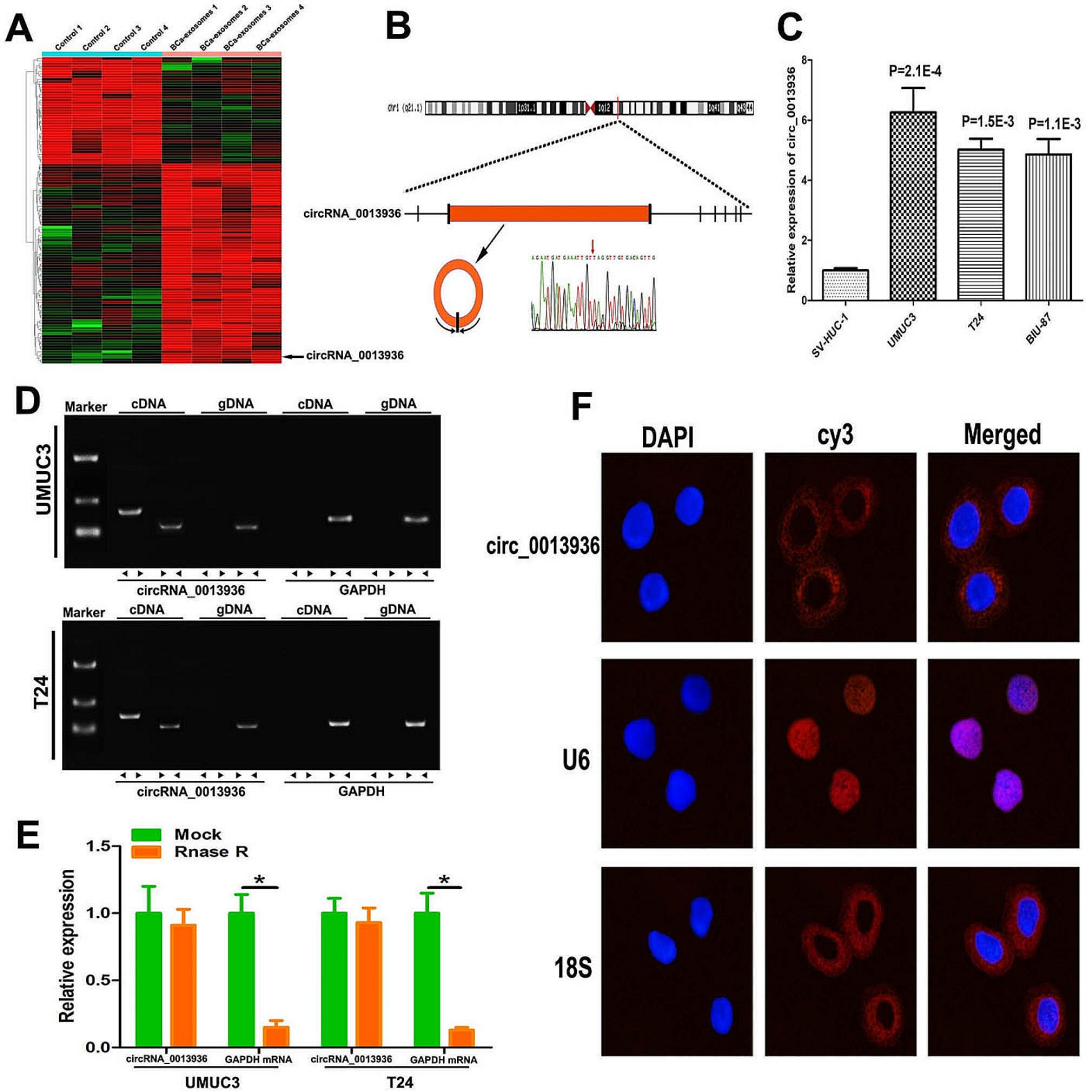
Identifcation and characterization of circRNA_0013936. (**A**) The heatmap showed the differentially expressed circRNAs (> 4-fold) in BCa-exosomes and SV-HUC-1-exosomes. (**B**) Schematic diagram of the composition of circ_0013936. (**C**) qRT-PCR was used to detect the expression of circ_0013936 in exosomes derived from three bladder cancer cell lines (T24, UMUC-3 and BIU-87) and normal urothelial cell (SV-HUC-1). (**D**) Sanger sequencing and agarose gel electrophoresis were used to verify the back splicing sequence and RT-PCR product of circ_0013936. (**E**) Detection of circRNA_0013936 expression by real-time PCR in T24 and UMUC3 cells treated with or without RNase R. (**F**) FISH confirmed circRNA_ 0013936 is mainly located in the cytoplasm. (*P < 0.05)

High-throughput sequencing was performed to identify differentially expressed circRNAs in BCa-derived exosomes. Figure 3A showed the differentially expressed circRNAs (> 4-fold). Among them, up-regulated circRNAs were more common than down-regulated circRNAs. We screened the up-regulated circRNAs with an average normal control read count of more than 100 and then sorted them by fold change. We selected ten most up-regulated circRNAs, and the details were listed in Supplementary Fig. 2A. The ten circRNAs were validated using qRT-PCR in 18 pairs of bladder cancer tissues and matched adjacent normal tissues (Supplementary Fig. 2B-K). According to the RNA-sequencing and qRT-PCR results, hsa_circ_0013936 was the up-regulated circRNA with the greatest expression difference (Supplementary Fig. 2C). Figure 3B showed the formation of circRNA_0013936. As shown by qRT-PCR, the expressions of circRNA_0013936 in exosomes derived from three types of BCa cells were significantly up-regulated compared with that in normal urothelial cell-derived exosomes (Fig. 3C). Further, we designed convergent primers and divergent primers to amplify circRNA_0013936. Using cDNA and gDNA (genomic DNA) from UMUC3 and T24 cell lines as templates, circRNA_0013936 was only amplified by divergent primers in cDNA, and no amplification product was observed in gDNA (Fig. 3D). By using qRT-PCR, we further confirmed that circRNA_0013936 was resistant to RNase R, while circRNA_0013936 was significantly reduced after RNase R treatment (Fig. 3E). FISH analysis indicated circRNA_ 0013936 was mainly located in the cytoplasm of bladder tumor cell (Fig. 3F).

### CircRNA_ 0013936 up-regulated FATP2 and down-regulated RIPK3 in PMN-MDSCs

**Fig. 4:**
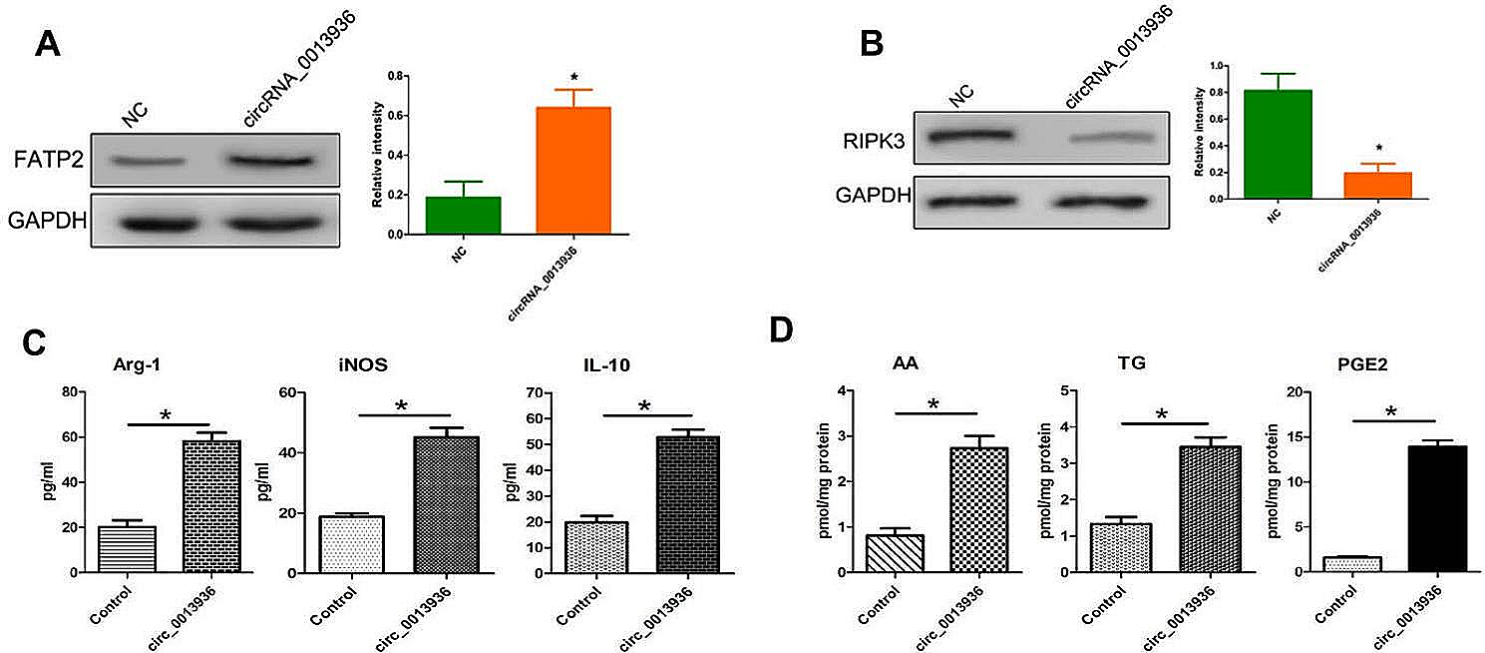
CircRNA_ 0013936 up-regulated FATP2 and down-regulated RIPK3 in PMN-MDSCs. (**A** and **B**) Western blotting detection of the expression of FATP2 and RIPK3 in PMN-MDSCs transfected with the cirRNA_0013936 mimics. (**C**) LC-MS detection of AA, PGE2, and TG in PMN-MDSCs. (**D**) ELISA detection of the immunosuppressive molecules (IL-10, INOS, Arg-1) in PMN-MDSCs. (* *P* < 0.05)

To confirm that circRNA_0013936 could up-regulate the expression of FATP2 and down-regulated the expression of RIPK3 in PMN-MDSCs, PMN-MDSCs were transfected with the circRNA_0013936 mimics. Western blotting analysis showed that the circRNA_0013936 mimics significantly up-regulated the expression of FATP2 and down-regulated the expression of RIPK3 in PMN-MDSCs (Fig. 4A and B). ELISA analysis showed that the circRNA_0013936 mimics promoted the synthesis of immunosuppressive cytokines (Arg-1, IL-10, iNOS) in PMN-MDSCs (Fig. 4C). LC-MS analysis showed that circRNA_0013936 mimics increased the levels of lipid metabolic molecules (AA, TG, PGE2) in PMN-MDSCs (Fig. 4D).

### CircRNA_ 0013936 functions as a sponge for miR-320a and miR-301b-3p

**Fig. 5:**
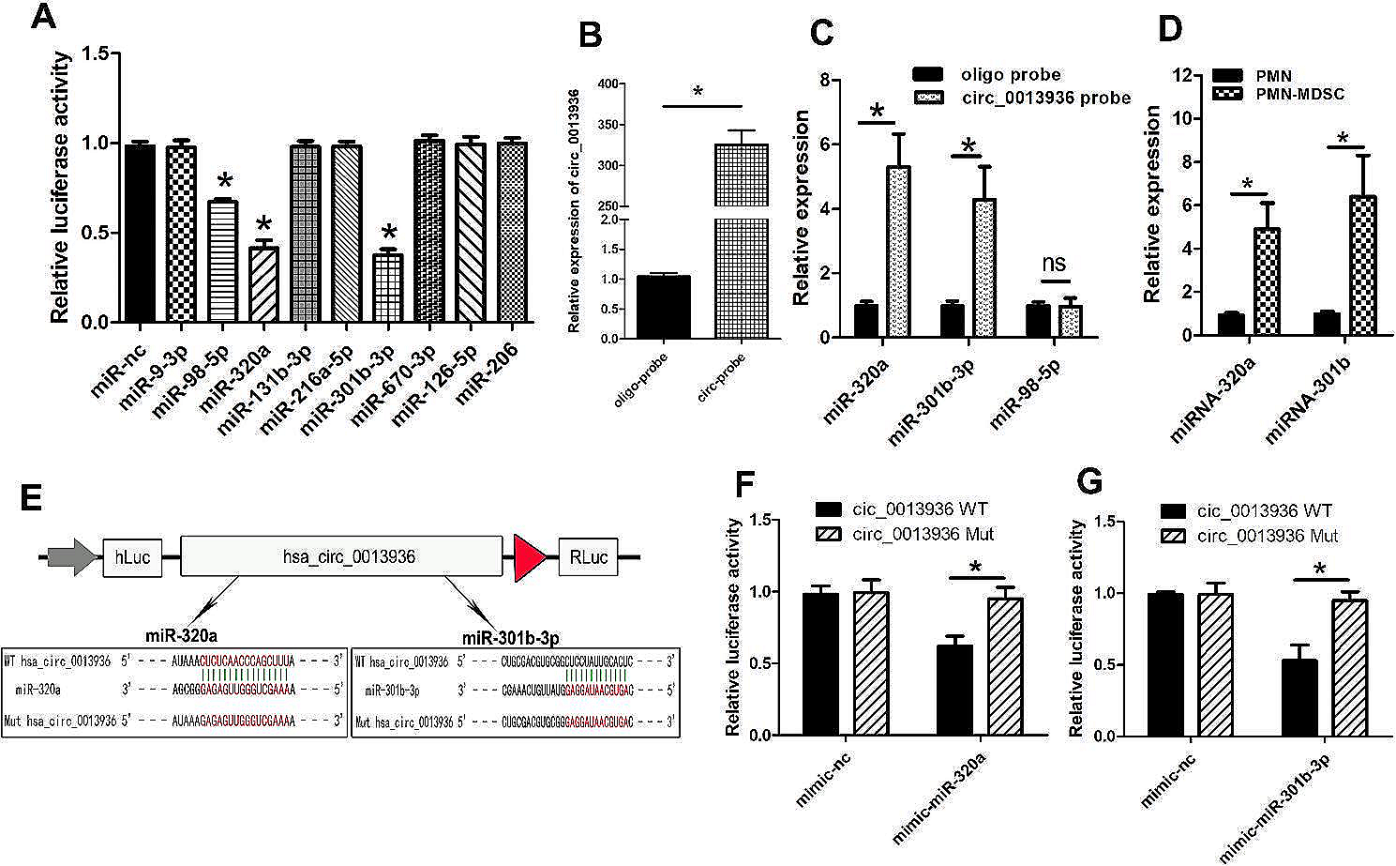
CircRNA_ 0013936 functions as a sponge for miR-320a and miR-301b-3p. (**A**) Evaluation of the effects of 9 candidate miRNAs on the luciferase activity of circ_0013936 using luciferase reporter gene assay. (**B** and **C**) The RNA pull-down assay was performed in PMN-MDSCs using circ_0013936 and negative probe. (**D**) The basic expressions of miR-320a and miR-301b-3p in PMN-MDSCs vs. PMNs. (**E**) Schematic diagram of mutant (Mut) or wild-type (Wt) circ_0013936 luciferase reporter vectors carrying miR-320a or miR-301b-3p binding sites. (**F** and **G**) The relative expressions of miR-320a and miR-301b-3p detected by qRT-PCR in 293T cells transfected with mutant (Mut) or wild-type (Wt) circ_0013936. (* *P* < 0.05)

To investigate whether circRNA_0013936 could sponge miRNAs in PMN-MDSCs, we identified nine of the most promising miRNAs with predicted score ≥ 90 by querying the CircInteractome database. We found three candidate miRNAs after analyzing the effects of nine candidate miRNAs on circRNA_0013936 luciferase activity through luciferase reporter assay (Fig. 5A). We designed a 3 ‘terminal-biotinylated circRNA probe to identify miRNAs that could bind to circRNA_0013936. As shown in Fig. 5B, the circRNA probe was used to pull down circRNA_0013936 in PMN-MDSCs. RIP circRNA_0013936 pull-down experiment found specific enrichment of circRNA_0013936, miR-320a, and miR-301b-3p, indicating that miR-320a and miR-301b-3p are the circRNA_0013936-associated miRNAs in PMN-MDSCs (Fig. 5C). Figure 5D shows the basic expressions of miR-320a and miR-301b-3p in PMN-MDSCs vs. PMNs. To further confirm whether miR-320a and miR-301b-3p could bind to circRNA_0013936, we conducted double-luciferase report assay in 293T cells. The results showed both miR-320a and miR-301b-3p mimic significantly reduced the luciferase activity of the WT-circRNA_0013936 (Fig. 5E-G). These results indicated that circRNA_0013936 functions as a sponge for miR-320a and miR-301b-3p.

### JAK2 is the direct target of miR-320a, and CREB1 is the direct target of miR-301b-3p

**Fig. 6:**
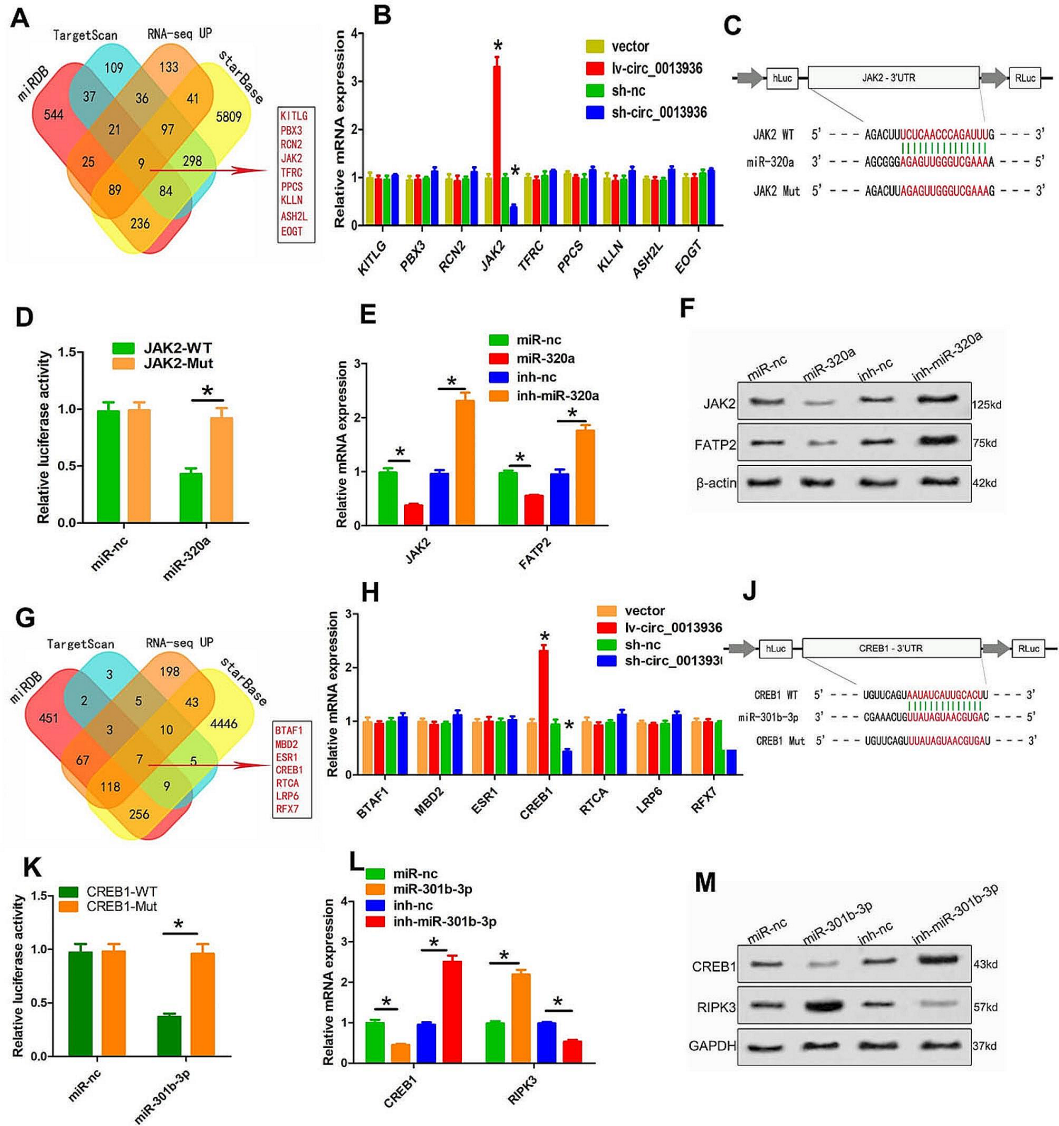
JAK2 is the direct target gene for miR-320a, and CREB1 is the direct target gene for miR-301b-3p. (**A** and **G**) Venn diagram showing the predicted target genes of miR-320a and 301b-3p. (**B** and **H**) qRT-PCR detected changes in mRNA levels of predicted targets overexpressed or silenced by circ_0013936. (**C** and **D**) Relative luciferase protease activity measured by luciferase reporter gene assay in 293T cells transfected with JAK2 (Mut or Wt) and miR-320a mimics. (**E** and **F**) Detection of JAK2 expression in PMN-MDSCs transfected with miR-320a inhibitors or miR-320a mimics using Western blotting and qRT-PCR. (**J** and **K**) Relative luciferase activity measured by luciferase reporter gene assay in 293T cells transfected with CREB1 (Mut or Wt) and miR-301b-3p mimics. (**L** and **M**) Detection of CREB1 expression in PMN-MDSCs transfected with miR-301p-3p inhibitors or miR-301b-3p mimics using Western blotting and qRT-PCR (*P < 0.05)

To further explore the underlying mechanism, we predicted the target genes of miR-320a and miR-301b-3p in Targetscan, miRDB, DIANA-microT and Starbase. Moreover, we analyzed the differential genes expression in PMN-MDSCs (circ_0013936-sh vs. circ_0013936-nc) by RNA-sequencing. Based on the results of RNA-sequencing and the prediction of target genes, we found that downstream target genes of miR-320a and miR-301b-3p have 9 genes and 7 genes, respectively (Fig. 6A and G). Then, qRT-PCR was performed to validate candidate genes. Among the genes containing conserved binding sites of miR-320a, JAK2 was up-regulated in PMN-MDSC-lv-circ_0013936 and down-regulated in PMN-MDSC-sh-circ_0013936 (Fig. 6B). Among the genes containing conserved binding sites of miR-301b-3p, CREB1 was up-regulated in PMN-MDSC-lv-circ_0013936 and down-regulated in PMN-MDSC-sh-circ_0013936 (Fig. 6H). In addition, dual luciferase reporter assay showed that miR-320a mimics significantly reduced the activity of wild-type of JAK2, but not the mutant of JAK2 (Fig. 6C–D), and miR-301b-3p mimics significantly reduced the activity of the wild-type of CREB1, but not the mutant of CREB1 (Fig. 6J–K). Furthermore, miR-320a/miR-301b-3p-overexpressing and miR-320a/miR-301b-3p-silencing cells were successfully constructed by transfecting with miR-320a/miR-301b-3p mimics or miR-320a/miR-301b-3p inhibitors. As shown in Fig. 6J-K and L-M, the expressions of JAK2 and CREB1 were significantly up-regulated or down-regulated at both mRNA and protein levels in PMN-MDSCs transfected with miR-320a/miR-301b-3p mimics or miR-320a/miR-301b-3p inhibitors.

### CircRNA_0013936 up-regulated FATP2 through circ_0013936/miR-320a/JAK2 pathway, and down-regulated RIPK3 through circ_0013936/ miR-301b-3p/CREB1 pathway

**Fig. 7:**
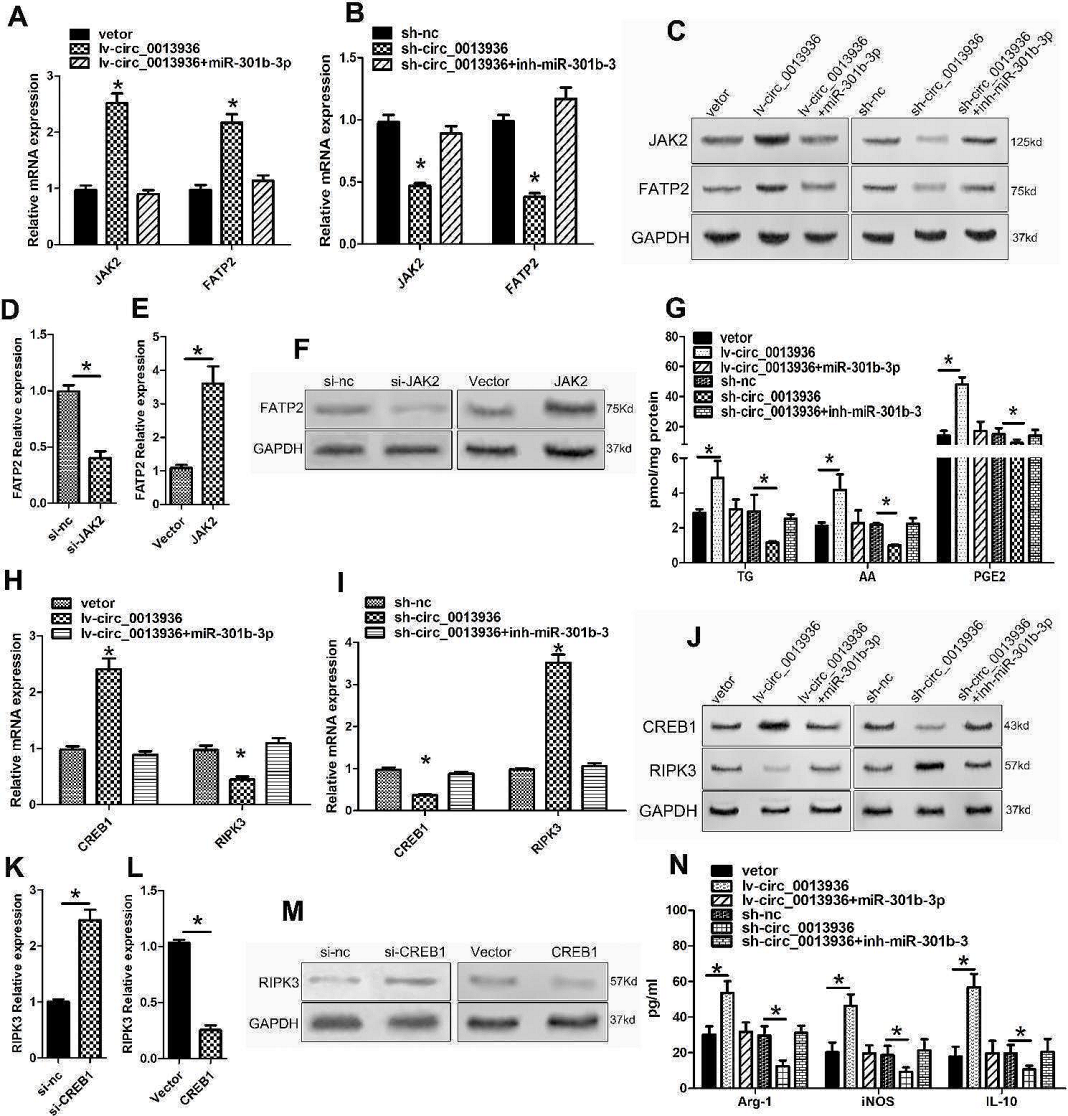
CircRNA_0013936 up-regulated FATP2 and down-regulated RIPK3 through the miR-320a/JAK2 and miR-301b-3p/CREB1 pathway in PMN-MDSCs. (**A**, **B** and **C**) The expressions of JAK2 and FATP2 in PMN-MDSCs after over-expressing or silencing circ_0013936 were detected by qRT-PCR and Western blotting. (**D**, **E** and **F**) The FATP2 expression in PMN-MDSCs after over-expressing or silencing of JAK2 were detected by qRT-PCR and Western blotting. (**G**) Detection of TG, AA, and PGE2 in PMN-MDSCs transfected with vector, lv-circ_0013936, mimic-miR-320a or mimic-nc using LC-MS. (**H**, **I** and **J**) Detection of CREB1 and RIPK3 expression in PMN-MDSCs after over-expressing or silencing circ_0013936 using Western blotting and qRT-PCR. (**K**, **L** and **M**) Detection of RIPK3 expression in PMN-MDSCs after silencing or over-expressing CREB1 using qRT-PCR and Western blotting. (**N**) Detection of immunosuppressive molecules (IL-10, INOS, Arg-1) in PMN-MDSCs transfected with vector, lv-circ_0013936, mimic-miR-301b-3p or mimic-nc using ELISA. (*P < 0.05)

CircRNA_0013936 increased the expression of FATP2 by promoting the expression of JAK2 in PMN-MDSCs, which was reversed by miR-320a mimics. While the silencing of circRNA_0013936 inhibited the expression of FATP2 by reducing the expression of JAK2, and miR-320a inhibitors could reverse the expression in PMN-MDSCs (Fig. 7A-C). CircRNA_0013936 inhibited the expression of RIPK3 by increasing the expression of CREB1 in PMN-MDSCs, which was reversed by miR-301b-3p mimics. While the silencing of circRNA_0013936 promoted the expression of RIPK3 by reducing the expression of CREB1, and miR-301b-3p inhibitors could reverse the expression in PMN-MDSCs (Fig. 7H–J). As shown by the results of qRT-PCR and Western blotting analysis, silencing of JAK2 or CREB1 significantly down-regulated FATP2 or up-regulated RIPK3 expressions in PMN-MDSCs, which was reversed by over-expressing JAK2 or CREB1 (Fig. 7D–F and K–M). CircRNA_0013936 promoted the production of cytokines derived from PMN-MDSCs (Arg-1, IL-10, iNOS, AA, TG, PGE2), which was reversed by miR-320a or miR-301b-3p mimics. While the silencing of circRNA_0013936 inhibited the production of cytokines derived from PMN-MDSCs, which was reversed by miR-320a or miR-301b-3p inhibitors (Fig. 7G and N). These results indicated that circRNA_0013936 regulated the expressions of FATP2 and RIPK3 by sponging miR-320a and miR-301b-3p.

### Exosomal circRNA_0013936 promotes suppressive immunity by up-regulating FATP2 through the circ_0013936/miR-320a/JAK2 pathway and down-regulating RIPK3 through the circ_0013936/ miR-301b-3p/CREB1 pathway in PMN-MDSCs

**Fig. 8:**
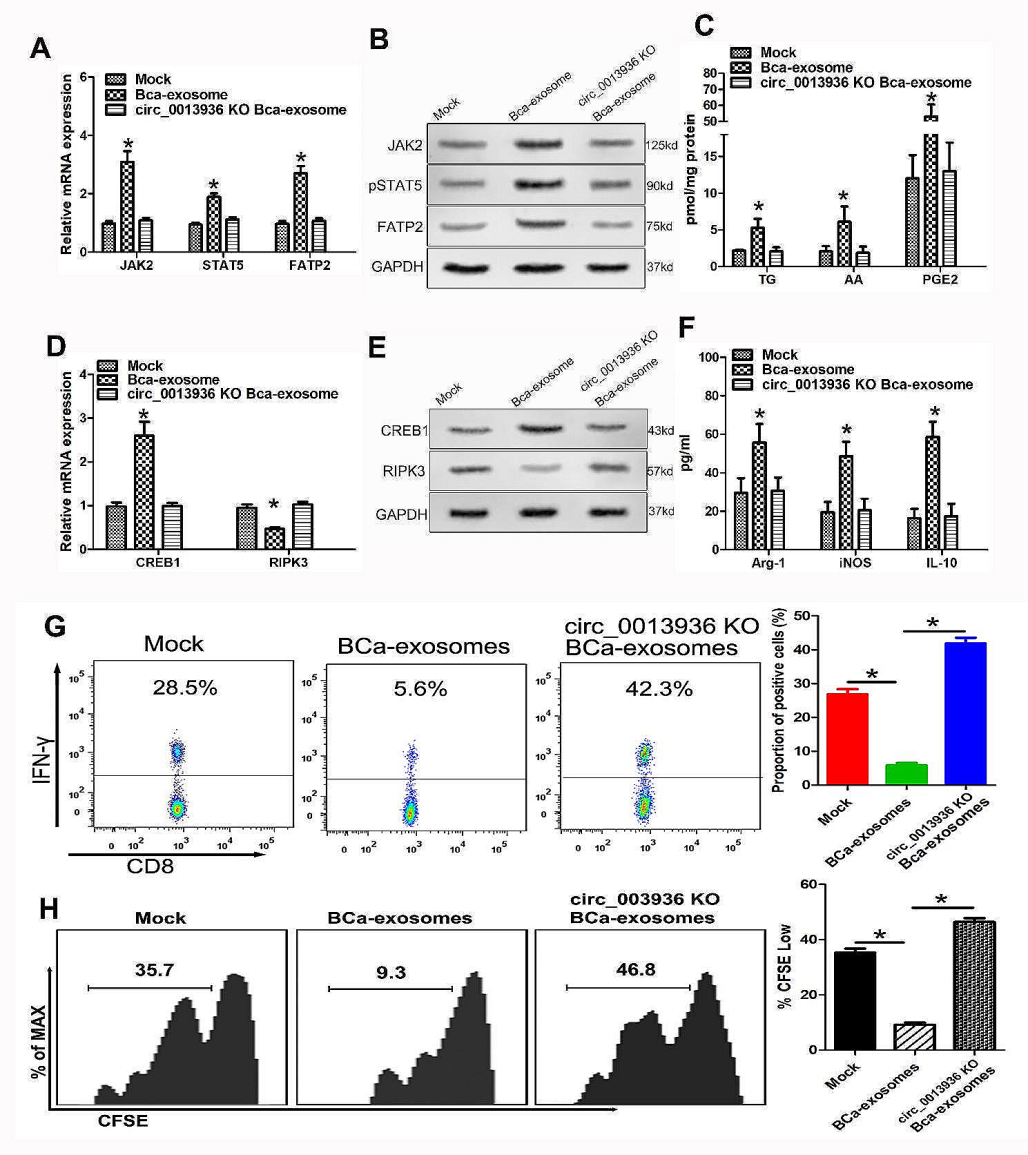
Exosomal circRNA_0013936 promotes suppressive immunity by up-regulating FATP2 through miR-320a/JAK2 pathway and down-regulating RIPK3 through miR-301b-3p/CREB1 pathway in PMN-MDSCs. (**A** and **B**) Detection of mRNA expression of JAK2, pSTAT5, and FATP2 in PMN-MDSCs co cultured with BCa derived exosomes or circRNA_0013936-KO-BCa derived exosomes using qRT-PCR and Western blotting. (**C**) Detection of TG, AA, and PGE2 in PMN-MDSCs using LC-MS. (**D** and **E**) Detection of mRNA expression of CREB1 and RIPK3 in PMN-MDSCs using qRT-PCR and Western blot. (**F**) Detection of immunosuppressive molecules (IL-10, INOS, Arg-1) in PMN-MDSCs using ELISA. (**G**) Flow cytometry was performed to evaluate the effect of PMN-MDSCs cocultured with BCa-derived exosomes or circRNA_0013936-KO-BCa-derived exosomes on the IFN-γ production of CD8^+^ T cells. (**H**) CFSE analysis was performed to evaluate the effect of PMN-MDSCs cocultured with BCa-derived exosomes or circRNA_0013936-KO-BCa-derived exosomes on the proliferation function of CD8^+^ T cells. (*P < 0.05)

To investigate whether BCa-derived exosomal circRNA_0013936 regulates the expressions of FATP2 and RIPK3 in PMN-MDSCs via miR-320a/JAK2 and miR-301b-3p/CREB1 pathways, circRNA_0013936 knockout bladder cancer cells generated using CRISPR-Cas9 genome-editing system were kindly provided by Guanxin Wang (Sun Yat-sen University, Guangzhou, China). We then detected the expressions of FATP2 and RIPK3 in PMN-MDSCs co cultured with BCa-exosomes or circRNA_0013936-KO-BCa-exosomes. As shown in Fig. 8A–B, BCa-derived exosomal circRNA_0013936 sponged miR-320a and then promoted the expression of FATP2 by activating JAK2/pSTAT5 pathway, while circRNA_0013936-KO-exosomes exhibited the opposite effects. Simultaneously, BCa-derived exosomal circRNA_0013936 sponged miR-301b-3p and then inhibited the expression of RIPK3 by activating CREB1 pathway, while circRNA_0013936-KO-exosomes exhibited the opposite effects (Fig. 8D, E). Ultimately, BCa-derived exosomal circRNA_0013936 promoted the production of immunosuppressive cytokines derived from PMN-MDSCs (Fig. 8C, F). To investigate whether BCa-derived exosomal circRNA_0013936 affected the functions of CD8^+^ T cells through regulating FATP2 and RIPK3 in PMN-MDSCs, CD8^+^ T cells were co cultured with PMN-MDSCs induced by BCa-derived exosomes or circRNA_0013936-KO-BCa-exosomes. Flow cytometry and CFSE analysis results showed that BCa-derived exosomes significantly inhibited the production of IFN-γ and the proliferation function of CD8^+^ T cells by regulating FATP2 and RIPK3 expressions, while circRNA_0013936-KO-BCa-exosomes exhibited the opposite effects (Fig. 8G–H).

## Discussion

The emergence of immunosuppressive cells in tumor tissue is a key factor for tumor cells to evade immune killing and treatment failure [20, 21]. In order to improve the efficacy of tumor immunotherapy, it is necessary to weaken the activity of these immunosuppressive cells [22, 23]. PMN-MDSC infiltrating the tumor microenvironment is an important immunosuppressive cell that leads to tumor progression [24]. PMN-MDSCs inhibit tumor immunity through various mechanisms, such as promoting the synthesis of various immunosuppressive cytokines [25–27], and inducing the maturation of Treg cells [28]. Weakening the immunosuppressive function of PMN-MDSCs helps improve the efficacy of tumor immunotherapy [29]. Therefore, further exploring the potential mechanism by which PMN-MDSCs exert immunosuppressive functions is of great significance.

In recent years, several studies have shown that FATP2 and RIPK3 play a crucial regulatory role in immune suppression led by PMN-MDSCs [8, 10]. Both FATP2 and RIPK3 enhance the inhibitory function of PMN-MDSCs by promoting the synthesis of PGE2, which plays an important role in the immunosuppressive function of PMN-MDSCs [30, 31]. In the study, we also found high infiltration of PMN-MDSCs in the tumor microenvironment, and the expression of FATP2 was significantly up-regulated in the tumor microenvironment, while the expression of RIPK3 was significantly down-regulated. PMN-MDSCs infiltration significantly correlated with FATP2 and RIPK3 expressions. After analyzing the clinical samples of human bladder cancer, we found that the expression of FATP2 in cancer stages III and IV was higher than that in stages I and II,and the expression of RIPK3 in cancer stages III and IV was lower than that in stages I and II. Through further research, we found that BCa-derived exosomes could up-regulate the expression of FATP2 and down-regulate the expression of RIPK3 in PMN-MDSCs. At present, the molecular mechanism underlying this phenomenon in the tumor tissue is still unclear.

FATP2 is one of the key molecules regulating lipid metabolism in PMN MDSCs [8]. Abnormal lipid metabolism is often present in the pathological activation of PMN-MDSCs [32]. In the tumor microenvironment, lipid accumulation was observed in PMN-MDSCs [33] and dendritic cells (DC) [34], and MDSCs associated with inhibitory immunity [35]. FATP2 has been reported to promote the immunosuppressive activity of PMN-MDSCs by enhancing the utilization of arachidonic acid and the synthesis of PGE2 [8]. Currently, phospholated STAT5 has been reported to be a key molecule regulating the expression of FATP2 [8], but the mechanism of regulating FATP2 is still not fully understood.

RIPK3, a key factor that regulates programmed cell necrosis, plays a crucial regulatory role in both inflammation and tumor development [36]. The expression of RIPK3 has been reported to be negatively correlated with tumor staging and is an effective prognostic indicator for various cancers [37]. As a study reported, the RIPK3-PGE2 circuit was an important regulatory mechanism for regulating the function of PMN-MDSCs [8]. PMN-MDSCs lacking RIPK3 exhibit stronger immunosuppressive activity [10]. Through NF-κ B-COX2-PGE2 signaling pathway, RIPK3 promotes the immunosuppressive activity of PMN-MDSC by promoting PGE2 synthesis. PGE2 inhibits the expression of RIPK3 through the cAMP/PKA signaling pathway, thereby promoting the expressions of NF-kB/COX-2 and Arg-1, ultimately forming the RIPK3-PGE2 circuit. Tumor cells activate the RIPK3-PGE2 circuit, which significantly promote the synthesis of PGE2 in PMN-MDSCs, ultimately enhancing the immunosuppressive function of PMN-MDSCs. Elucidating the underlying molecular mechanism can help overcome PMN-MDSCs mediated inhibitory immunity and may provide effective therapeutic targets for tumor immunotherapy.

Currently, there are no studies reporting the mechanism by which BCa-derived exosomes regulate the expressionss of RIPK3 and FATP2 in PMN-MDSCs. CircRNA has the characteristics of widespread expression and strong regulatory effects, making it a functional biomarker and therapeutic target for various diseases [38]. However, although there have been many studies on circRNAs in tumor regulation, their roles in the progression of BCa still need further clarification. Here, we report for the first time a new circRNA, named circRNA_0013936, highly expressed in BCa-derived exosomes. In addition, a series of experiments showed that circRNA_0013936 promoted the expressions of RIPK3 and FATP2 in PMN-MDSCs, while silencing the circRNA_0013936 has the opposite effect. CcircRNA contains many potential miRNA responsive elements, indicating that circRNA can act as a miRNA sponge and exert its regulatory role through the circRNA-miRNA-mRNA axis [39]. In this study, we found that circRNA_0013936 was highly expressed in BCa cells, especially in the cytoplasm of tumor cells. Therefore, we speculated that circRNA_0013936 might also regulate the function of PMN-MDSCs through miRNA sponge.

Through bioinformatics analysis, miRNA pull-down assay, and luciferase assay, we found that circRNA_0013936 could sponge miR-320a and miR-301b-3p. MiRNA has been extensively demonstrated to play an important regulatory role in the occurrence and development of tumor cells by reducing the inhibitory effect of its target genes [40]. In this study, based on mRNA expression profiles, bioinformatics analysis, and analysis of dual luciferase reporter genes, JAK2 may serve as the direct target for miR-320a, and CREB1 may serve as the direct target for miR-301b-3p. Molecular functional experiments showed that circRNA_0013936 sponged miR-320a and miR-301b-3p in PMN-MDSCs, thereby reducing the inhibitory effects of miR-320a on JAK2 and miR-301b-3p on CREB1, and ultimately regulating the expressions of FATP2 and RIPK3 in PMN-MDSCs.

In summary, we found that BCa-derived exosomal circRNA_0013936 up-regulated FATP2 by sponging miR-320a through circRNA_0013936/miR-320a/JAK2 pathway and down-regulated RIPK3 by sponging miR-301b-3p through circRNA_0013936/miR-301b-3p/CREB1 pathway, and ultimately promoted the synthesis of immunosuppressive molecules, which severely inhibited the functions of CD8^+^T cells and the secretion of IFN-γ by CD8^+^T cells. Our research results indicate that circRNA_0013936 is expected to become an effective therapeutic target for BCa. These findings provide a theoretical basis and new ideas for the clinical treatment of bladder cancer.

### Electronic supplementary material

Below is the link to the electronic supplementary material.

Supplementary Material 1

Supplementary Material 2

## Data Availability

All data generated or analyzed during the current study are included in this published article.

## Abbreviations

PMN-MDSCs: multinucleated myeloid-derived suppressor cells
BCa: bladder cancer
RIPK3: receptor-interacting protein kinase 3
FATP2: fatty acid transporter protein 2
PGE2: prostaglandin E2
Arg-1: arginase 1
ROS: reactive oxygen species
iNOS: inducible nitric oxide synthase
