# Supplementary figures and images for: Bladder-cancer-derived exosomal circRNA_0013936 promotes suppressive immunity by up-regulating fatty acid transporter protein 2 and down-regulating receptor-interacting protein kinase 3 in PMN-MDSCs
Source: Mol Cancer. 2024 Mar 9;23:52. doi: 10.1186/s12943-024-01968-2 (PMC10924381; doi:10.1186/s12943-024-01968-2)

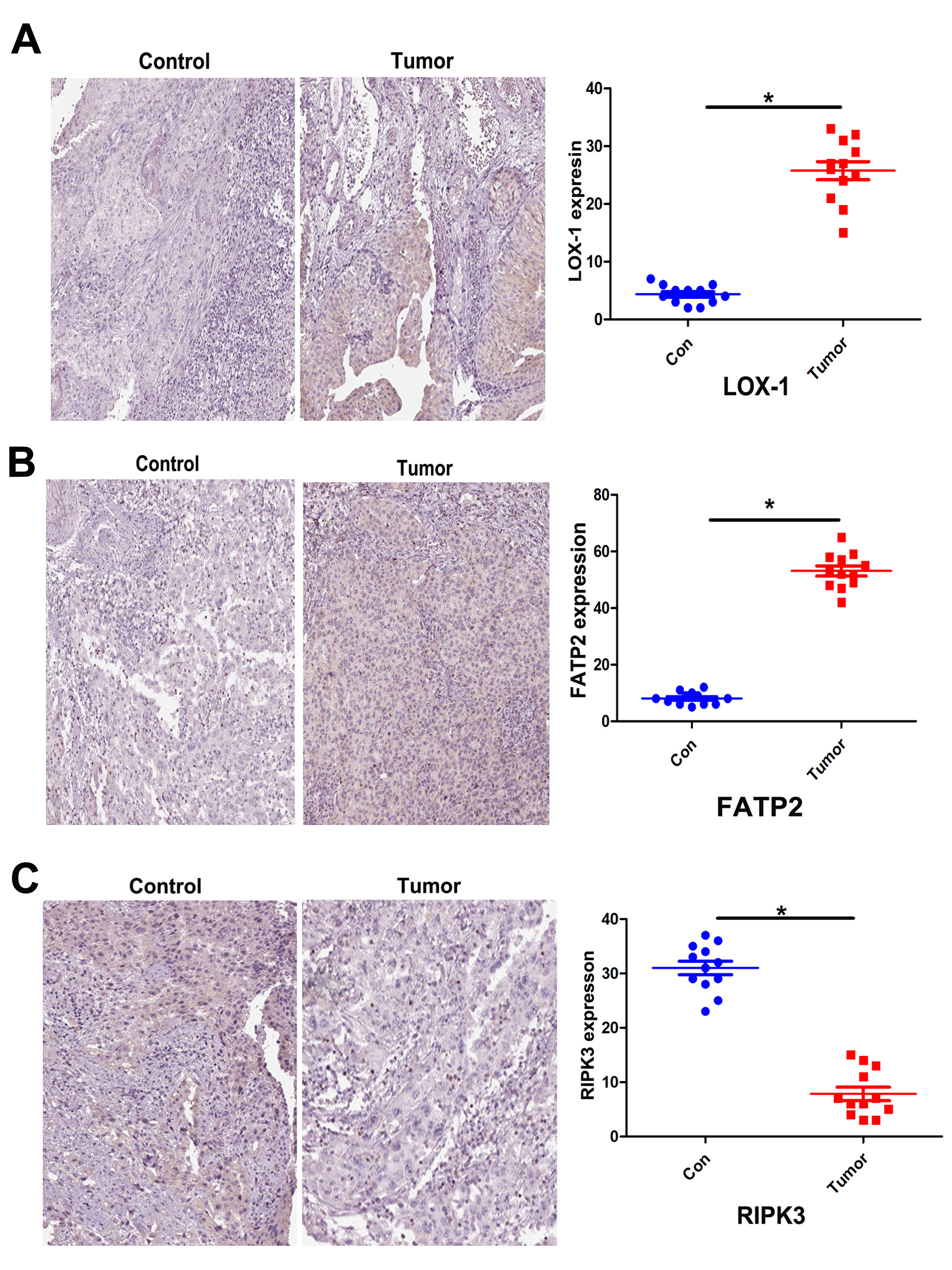

Supplement: Supplementary file 1 — Supplementary Material 1 [file 12943_2024_1968_MOESM1_ESM.jpg]

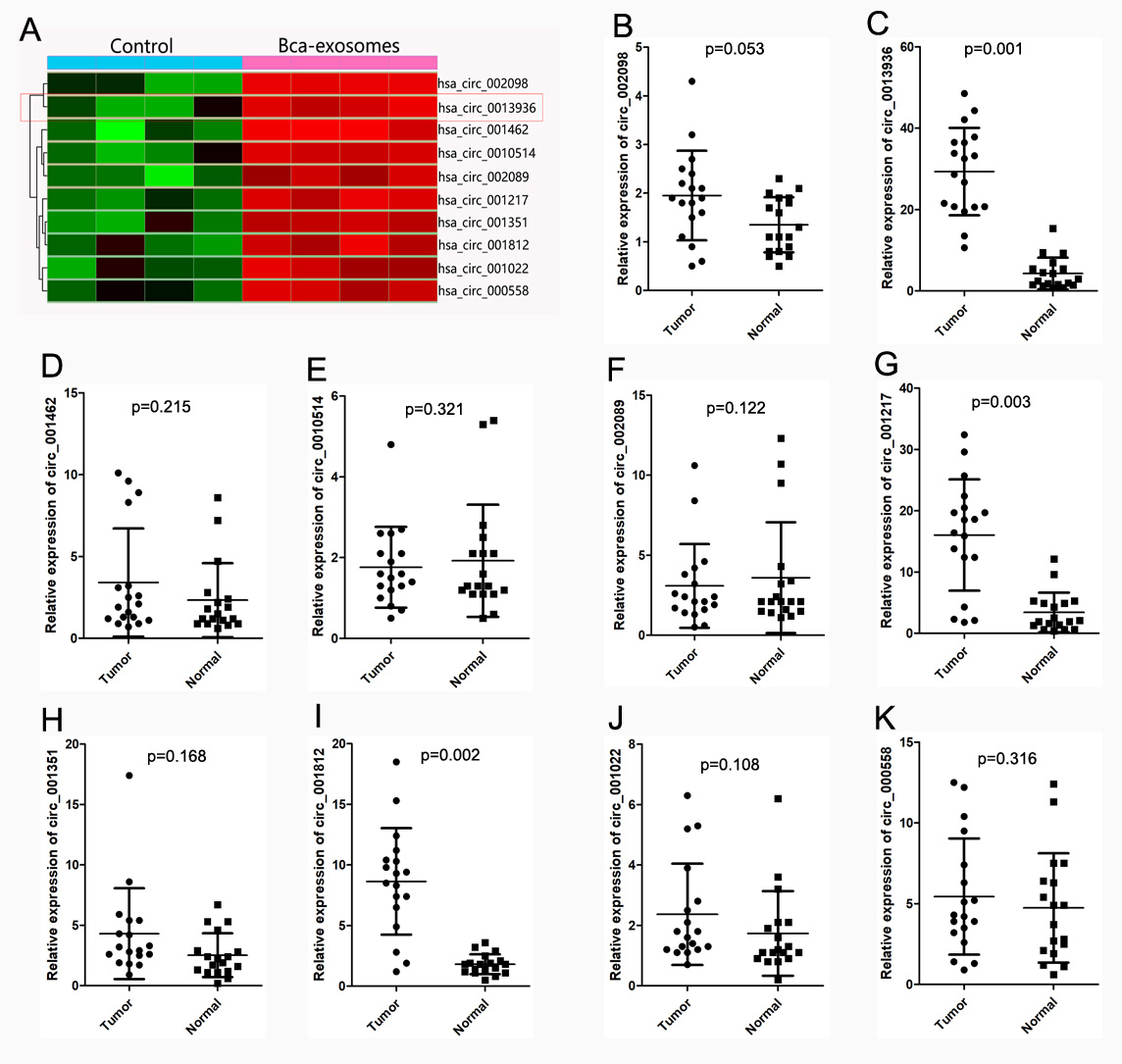

Supplement: Supplementary file 2 — Supplementary Material 2 [file 12943_2024_1968_MOESM2_ESM.jpg]
